# Tropical Coastal Land-Use and Land Cover Changes Impact on Ecosystem Service Value during Rapid Urbanization of Benin, West Africa

**DOI:** 10.3390/ijerph18147416

**Published:** 2021-07-11

**Authors:** Damien Sinonmatohou Tiando, Shougeng Hu, Xin Fan, Muhammad Rashid Ali

**Affiliations:** 1 Department of Land Resource Management, School of Public Administration, China University of Geosciences, Lomo Road 388, Wuhan 430074, China; damientiando90@gmail.com (D.S.T.); 2201510301@cug.edu.cn (X.F.); 2 Registrar Office, Confidential Branch, Government College University Lahore, Katchery Road, Lahore 54000, Pakistan; dr.mrashid@gcu.edu.pk

**Keywords:** land-use and land cover changes, ecosystem services value, urbanization, land-use proxy-based method, Benin

## Abstract

West African coastal areas including the Beninese coastal zones have undergone an intensification of socio-economic activity in the last few decades that has been strongly driven by the effects of rapid urbanization. This has led to land-use and land cover changes that represent threats to the sustainability of various ecosystem functions. Such dynamics of land use and land cover changes pose challenges to coastal zone management. Correct assessment is vital for policymakers and planners to ensure efficient and sustainable use of the coastal ecosystem services, and it remains crucial to achieving sustainable coastal zone management. This study examines changes in land-use and land cover (LULC) and their impacts on ecosystem services value (ESV) fluctuations in the tropical coastal region of Benin, West Africa. We employed Globe Land 30 image data for the years 2010 and 2020, and the ESV fluctuations during the study period were evaluated using the benefit transfer approach (BTA) with corresponding local coefficients values and the GIS techniques. The results reveal that (1) in the current urbanizing coastal area, the LULC types have changed significantly, with obvious reductions in forest land and waterbodies and a considerable increase in artificial surfaces; (2) the total ESV decreased by 8.51% from USD 7.1557 million in 2010 to USD 6.5941 million in 2020; (3) the intensity of LULC in the coastal region has increased over the last 10 years; (4) regions with high land-use intensity have a high rate of ESV change; and (5) provisioning services are the greatest contributors of ESV (51% in 2010; 41% in 2020), followed by supporting services (37% in 2010; 35% in 2020) and regulating services (25% in 2010; 30% in 2020). Uncontrolled changes in LULC from forest land and waterbodies are the main causes of the loss in total ESV, necessitating urgent measures to improve the coastal ecosystem sustainability through effective planning and policies.

## 1. Introduction

The concept of ecosystem services brings challenges in evaluating and balancing between sustainable socio-economic development and natural resources protection. Scientific assessment of the concept has concerned scholars around the world since the last decade, and it has recently been discussed in the context of Sustainable Development Goals (SDGs), which call for synchronized international efforts towards a more resilient and rational use of ecosystem services [1,2]. However, integrating ecosystem services and socio-economic characteristics into decision-making processes remains a challenge. Ecosystem services represent goods and services provided either directly or indirectly by the functions of the ecosystem on which human existence depends [3,4]. For instance, the provision of ecosystem services can directly affect changes in the extent and composition of forest, wetlands, river, and agricultural land.

The assessment of ecosystem services covers the loss or benefit costs of preserving a given amount or quality of an ecosystem service and is an integral part of the conservation decision-making process [5,6]. Two methods of evaluating ecosystem services have been widely used to estimate the ESV: First, the contingent economic approaches concerning market prices, travel cost, production approaches, and opportunity costs [7,8]. The second technique is the land-use proxy-based method, or the benefit transfer approach (BTA). The BTA combines remote sensing and GIS technologies to estimate the ESV and map the services’ distributions [9,10]. The BTA has been extensively used by scholars due to the lack of primary data and limited financial resources [11]. The BTA offers immediate information to decision-makers on various aspects of policy actions and strategies for the sustainable management of land resources, and it has been widely used by researchers to assess the values of ecosystem services at different spatial scales, such as countries, provinces, cities, urban agglomerations, and watersheds [6,12].

Coastal zones are the transitional areas between sea and land and are an important geographic zone both in terms of resources and human habitation [13]. They are environmentally complex and sensitive, with coastal ecosystems being among the most productive on Earth [14]. They sustain the stability of coastal aspects, represent potential hazards, and enable sustainable economic development [15,16]. Nevertheless, coastal ecosystems nowadays fall among the most affected ecological areas due to the continuous intensification of anthropological activity, including not only coastal developments such as land reclamation, but also pollution from upland agriculture and industry [16,17]. Coastal ecosystem services are more liable to suffer irreversible damage caused by human activities than that caused by biophysical drivers [18,19]. These changes are pronounced in West African coastal areas, particularly in low-income coastal areas, with LULC changes accountable, directly or indirectly, for the degradation of coastal zones, their ecosystems, and their capacity to produce sustainable resources [20,21]. Socio-economic activities, such as urbanization associated with coastal population growth, traditionally occur parallel with LULC changes (mainly from forests and grassland to farmland and built-up areas, and the conversion of coastal wetland into farmland [22]. Globally, the conversion of natural ecosystems into agricultural land increases food production, housing, and other goods but also may generate an accompanying decline in the provision of several ecosystem services. It has been estimated that 40% of the agricultural land of the Earth’s surface area has been converted by forests being cropped, due to the increasing human population and economic development [23]. Consequently, the ecosystems sustained by these natural areas are lost as they shrink.

Changes in LULC are among the main driving factors of ESV fluctuation [23,24]. LULC changes impact the status and integrity of an ecosystem, which affects its functions and the services it provides to humans [25]. The assessment of ESV from the perspective of LULC change has significance in evaluating the impacts of land-use changes on a coastal ecosystem [26]. Consequently, deepening our understanding of the importance of coastal development decisions and the consumption of coastal resources will help us to better use ecosystem services.

Benin is a West African country located in the Northern hemisphere. It is bounded by Togo to the west, Burkina Faso and Niger to the north, Nigeria to the east, and Bight of Benin to the south. The coastal zones of Benin constitute important coastal ecosystems containing various coastal wetlands, mainly composed of mangroves, that are important for the maintenance of biodiversity and also for their role in sustainable socio-economic development [27]. However, the current strategies of coastal zone management are dealing with LULC changes, as well as the degradation of the natural coastal ecological environment [28]. Under the current situation, though, the assessment of ESV related to LULC changes in Benin’s tropical coastal areas has been relatively scarce and has been limited to several small coastal areas comprising a few districts of Benin [28,29], leaving the understanding of LULC within the broad coastal areas limited. To date, there has been no assessment of the impacts of tropical LULC on ESV across the coastal zones. There is still a lack of fine resolution spatial description of the effects of human activities on ESV. Additionally, the method of deriving ESV from local coefficients modified for the study area has limitations. To overcome this, this study covers the whole tropical coastal region of Benin and uses the benefit transfer method and satellite imagery data to analyze LULC changes in the tropical coastal region of Benin within a ten year period. We provide a scientific basis for local and regional actions and policymaking strategies that can be employed to ensure the sustainable management and rational use of the coastal ecosystems.

## 2. Materials and Methods

### 2.1. Study Area

**Figure 1:**
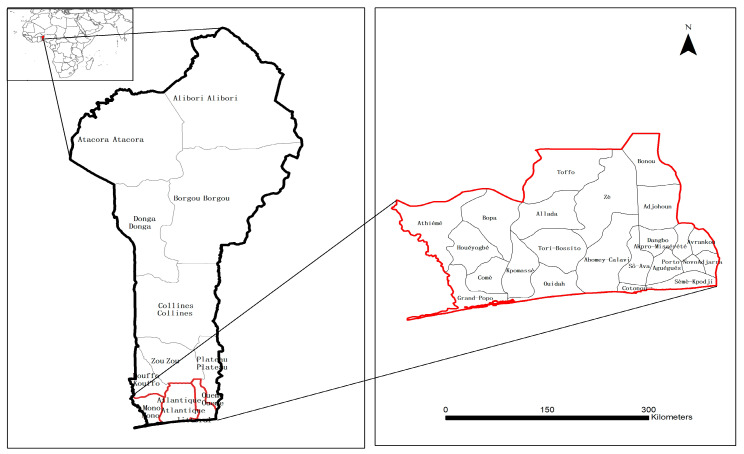
The study area of the tropical coastal zones of Benin.

The coastal zone of Benin (Figure 1) spans between 1°35′ and 7°30′ eastern longitude from Togo in the west to Nigeria in the east and between 6°20′ and 7°30′ northern latitude. It covers 125 square kilometers corresponding to approximately 10.5% of the total country’s territory [28]. The coastal area is densely populated, and the current number of inhabitants is estimated at 3.66 million, with a yearly growth rate of 2.8% compared to 3.5% for the whole country [29]. The coastal zone’s environment consists of valuable ecosystems, including rivers, farmland, forest land, and various wetlands, which favor the development of several socio-economic activities such as sea transport, fisheries, tourism activities, and mineral and industrial operations. The Beninese coastal zones contribute more than 70% of the country’s GDP [30]. Since the beginning of the 1990s, the intensification of socio-economic activity along the coastal zones has led to rapid urbanization and the growth of the coastal population. Recent trends have led to LULC changes and the degradation of natural coastal resources, as well as losses of biodiversity, to which is added natural threats such as climate change, coastal erosion, marine submersion, rising temperatures, etc. [29,30].

### 2.2. Satellite Imagery Data and Classification

In this study, the LULC data for 2010 and 2020 (Global Land 30 products with 30 m spatial resolution) were obtained from the China Environmental Disaster Reduction Satellite [31,32]. According to third-party inspection, the product classification accuracy is 83% [32]. The original dataset contained ten categories of LULC. Given the current LULC management features of the study area, we have selected seven categories of LULC (Table 1) including: farmland, forest land, grazing land, wetland, shrubland, waterbodies, and artificial surfaces.

To determine the suitability of the datasets for LULC change analysis in the study area, we conducted an accuracy checking of the datasets using high-resolution Google Earth images (https://www.google.com/earth/, accessed on 8 August 2020). A total of 400 points (including 50 points for each LULC type) distributed over the study area were acquired through a random sampling method for both periods. These points were overlaid on top of the Google Earth satellite images, and the LULC type of each point was validated using the Google Earth images temporally around the Globe Land 30′s production year. The LULC datasets for each year were used as proxies for the measurement of the ESV, and the corresponding area in hectares was assessed and presented in a raster in the GIS [33].

### 2.3. Statistics Analysis

#### 2.3.1. Comprehensive Land-Use Dynamic

Land-use dynamic degree, also known as the land use change rate index, is mainly used to calculate the quantitative value of land use type change, and can also be used to estimate the land use change trend and the change speed in a few years. In this study, the land-use dynamic degree was introduced to quantify the variation of land use during our study period [34,35]. A dynamic degree with a value over zero means that this LULC type has increased compared to other types. However, if the value is below zero, it shows that this LULC type is being depleted. Additionally, a transition matrix was generated to capture the multidirectional change between the LULC types.

#### 2.3.2. Land-Use Intensity Analysis

Land-use intensity indicates the degree of the interference of human activities on lands. The coastal zones of Benin are experiencing urbanization, with the intensification of human activities. The land-use intensity analysis expresses the comprehensive impacts of human activities on the variation in LULC during urbanization and the conversion of land for coastal development [24,36]. The land-use intensity was evaluated via the equation below:

I = ∑ (L_i_ P_i_) × 100% (1)

where Ⅰ is the land-use intensity comprehensive index of the study area related to human activity, L_i_ is the level or stages index of land-use intensity of LULC type i, and P_i_ is the quantity of the LULC type. With reference to Cao, Li et al. [24,33], considering the current characteristics of land-use management in the study area, and according to the natural balance of land under the influence of social factors, specifically based on the degree of human disturbances in our study area which may differently affect various land types, the comprehensive index of land-use intensity has been divided into five levels (Table 2).

Additionally, in order to understand the spatial characteristics of changes in land-use intensity we transformed the land use vector map into a grid dataset of land use at 30 m scale firstly, and then calculated the land use intensity comprehensive index at 30 m scale by Equation (1). Secondly, we created a 30 m fishnet; then, the land-use vector map was combined with the fishnet to calculate the area of each LULC type in each grid cell. Finally, we recorded the area of a specific land-use type with a grid data layer at the 30 m scale.

#### 2.3.3. Ecosystem Services Value Assessment

The present assessment of ESV in the tropical coastal region of Benin used a benefit transfer approach (BTA) based on the modified local value coefficients proposed by Kindu, Temesgen et al. [6,37] (See Table 3 and Table 4). These value coefficients have been adjusted from the value coefficients proposed by Costanza et al. [20,38], and operate via the adaptation of existing values from one area to estimate the ESV of another similar area [22,38]. These coefficients were applied because they have been properly established for low-income countries’ coastal areas, including Benin’s [22,38]. We used the most representative biomes as the proxy for different land uses in the study area. Cropland biomes were used as a proxy for farmland and aquaculture land, tropical forest biomes were used for forests, urban biomes for built-up land, and grasslands and shrublands have the same equivalent biome: rangelands.

The ESV of each LULC category has been evaluated by multiplying the area of each LULC category by the coefficient value of the biome used as the proxy for that category (Table 3) as follows:

ESV = ∑ (A_k_ × VC_k_) (2)

where A_k_ is the area of LULC type K, and VC_K_ is the value coefficient (USD/ha/year). We also estimated the value provided by individual ecosystem functions within the study area using the following equation:

ESV_f_ = ∑ (Ak × VC_f_) (3)

where A_k_ is the area of LULC type K, and VC_f_ is the value coefficient of the function f (USD/ha/year).

#### 2.3.4. Coefficient of Sensitivity (CS)

There are several limitations of the methodology of benefit transfers which we have adopted in this study. Different biomes were used as proxy for different land uses, although they may not match appropriately because there are so many uncertainties about the ESV of the different land use types. Due to these uncertainties of proxy values, a coefficient of sensitivity was analyzed using the standard economic concept of elasticity, i.e., the percentage change in the output for a given percentage change in an input. The coefficient of sensitivity (CS) or coefficient of elasticity (CE) is used to determine the sensitivity and robustness of coefficients in the analysis of ecosystem services [37,39]. The CS was determined via the equation below.

CS = VC_i k_ × (A_k_/ESV_i_) (4)

With VC and A_k_, respectively, represent the value coefficient and the size of land-use type K, while ESV_i_ represents the initial value of ESV. The higher the CS value, the more important the corresponding land-use type is to the total ESV.

## 3. Results

### 3.1. Variation in Land-Use and Land Cover from 2010 to 2020

**Figure 2:**
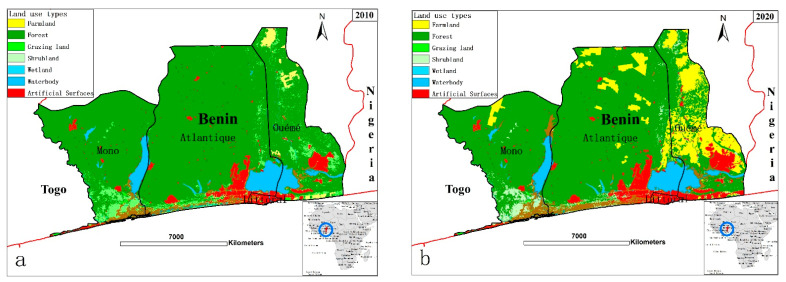
Spatial distribution of land-use and land cover type in Benin tropical coastal zones: (**a**) Spatial distribution in 2010; (**b**) Spatial distribution in 2020.

The variation in each LULC type over our study period was analyzed via the superposition of the land data, and we generated spatial distribution maps in 2010 and 2020 (Figure 2). In 2010, forest land had the greatest distribution in the study area (79.1%), followed by waterbodies (5.7%), and grazing land (5.6%). Artificial surfaces covered 4.7% and farmland covered 1.2%. In 2020, forest land occupied 68.3%, while artificial surfaces occupied 6.7%, and farmland 11.6%. The greatest reduction was recorded in forest land (10.2%), followed by grassland (2.1%). From the spatial distribution of Figure 2, farmland was mainly concentrated in the eastern and south-eastern coastal regions, including the Ouémé Valley coastal wetland, while the forest was mainly located in the south-eastern coastal region. Farmland showed the greatest increase in change rate (63%), and artificial surfaces showed the second highest rate (10%), with their corresponding areas increasing from 302.27 ha in 2010 to 407.80 ha in 2020, during which time the coastal zones were undergoing urbanization (see Table 5).

### 3.2. Spatial-Temporal Features of Land Use Change and Intensity from 2010 to 2020

**Figure 3:**
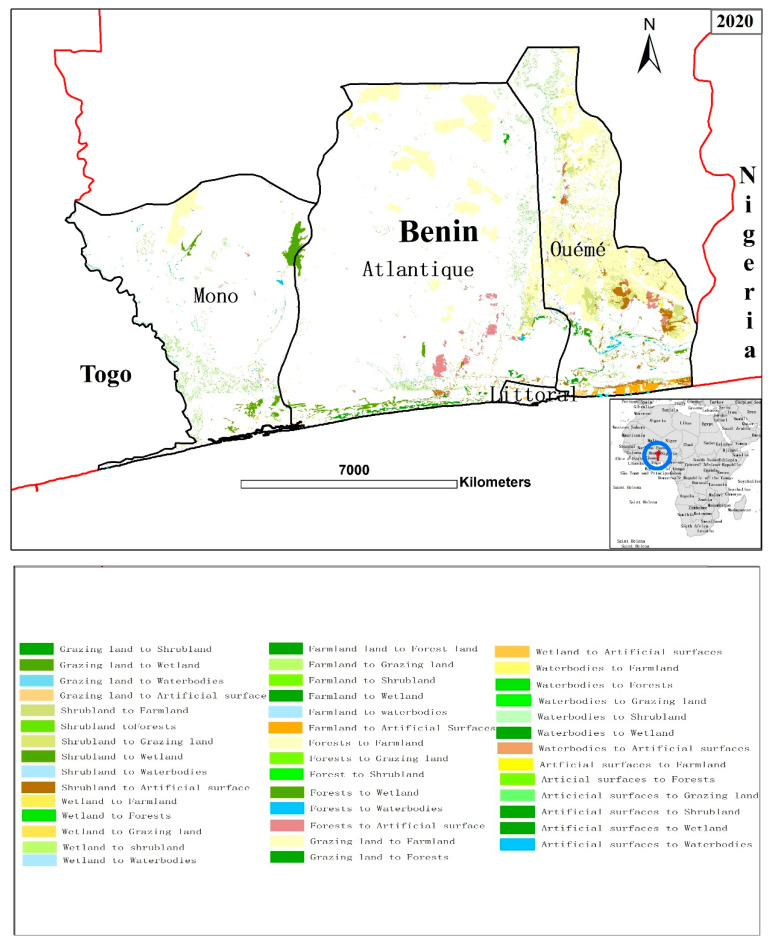
Transition patterns of land-use and land cover conversions in Benin.

**Figure 4:**
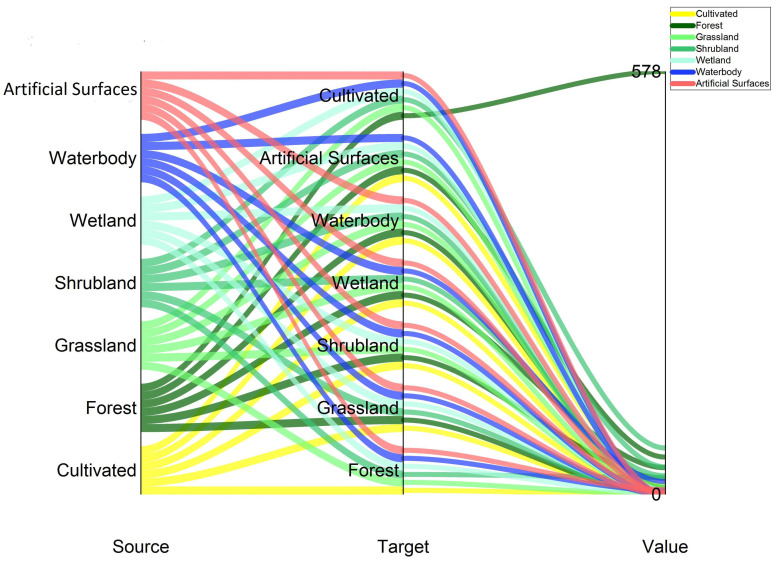
Flow chart of land-use and land cover change directions.

With the support of the ArcGIS software spatial block statistics tool, the conversion matrix of LULC types (Figure 3) has been produced in order to understand the structural transformation process that caused land use change between 2010 and 2020 and also the flow (Figure 4) of various land use types. From the quantitative results of the conversion of different land-use types (Table 6). The diagonal values (in bold) represent the area of each LULC type that remained stable from 2010 to 2020 while the off-diagonal values represent the change area.

It can be seen that farmland and artificial surfaces have increased while the other land-use types decreased during our study period. The expansion of farmland (643.66 km^2^) was mainly produced via conversion from forest (577.54 km^2^) and shrubland (63.63 km^2^). In the same time period artificial surfaces (336.21 km^2^) expanded, mainly converted from farmland (12.23 km^2^), forests (51.24 km^2^), and shrubland (37.82 km^2^). Wetland (336.21 km^2^) expanded mainly converted from forest (36.66 km^2^) and shrubland (25.51 km^2^).

**Figure 5:**
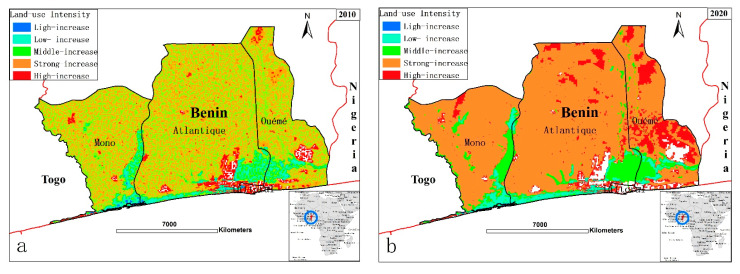
Spatial distribution of land-use intensity from 2010 to 2020 in Benin tropical coastal region. (**a**) Spatial patterns in the year 2010, (**b**) Spatial patterns in the year 2020.

By comparing the two maps of land-use intensity (Figure 5), it is obvious that the land-use intensity index has increased during the last 10 years in our study area. With the increased rate of coastal development and urbanization, the intensity indicators of the different LULCs have increased. Much greater high-intensity components have been recorded in the eastern industrial coastal zones of Cotonou and Seme-Podji, whereas it was lower in the western coastal region of Grand-Popo.

### 3.3. Changes of Ecosystem Services Value in Benin Tropical Coastal Region

**Figure 6:**
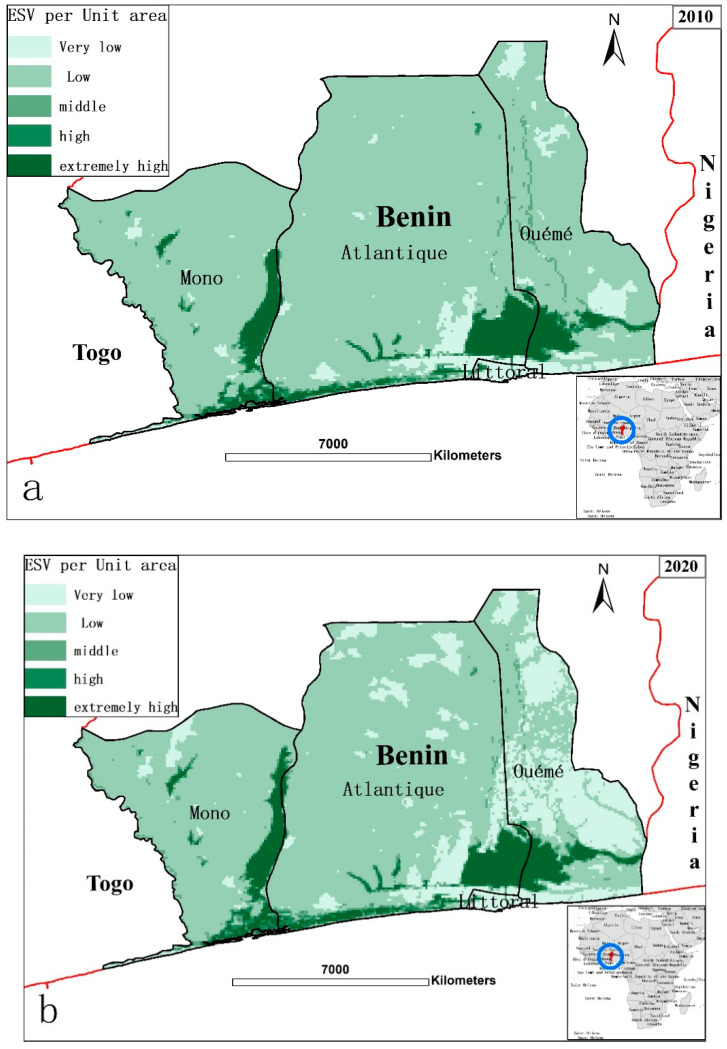
Spatial patterns of ESV in the tropical coastal area of Benin. (**a**) Spatial patterns in 2010, (**b**) Spatial patterns in 2020.

Changes in the ESV of each LULC type in our study area were assessed based on the modified ecosystem services value coefficient, such as those used by Kindu et al. [6]. The results are given in Table 7. The total ESV in our study area had decreased by −7.85% from USD 7.1557 million in 2010 to USD 6.5941 million in 2020. Forests made the greatest contribution to ESV (73% in 2010; 68% in 2020), followed by waterbodies (15% in 2010; 16% in 2020) and wetland (4% in 2010; 3% in 2020). Grazing land made the smallest contribution to ESV, whereas forests and waterbodies were the greatest contributors to total ESV, accounting for more than 80% (Figure 6).

By using the spatial analysis tools of ArcGIS, the ESV of the different units was calculated and characterized into five levels as follows: very low (<USD 10.000/ha), low (USD 10,000/ha~USD 30,000/ha), middle (USD 30,000/ha~USD 50,000/ha), high (USD 50,000/ha~USD 70,000/ha), and extremely high (>USD 70,000/ha). The spatial distribution patterns of ESV for each component are shown in Figure 6. Noticeably, most units in the study area were converted from high levels to low levels. Units with an extremely high ESV value were mainly contributed by the coastal wetland ecosystem of Ouémé Valley and the coastal waterbodies of Lac Nokoue and Porto-Novo Lagune in the eastern coastal zone. The ESV of the Seme industrial coastal area became significantly narrower due to rapid urbanization and was gradually converted into a low or very low ESV. Units with a low ESV were mainly farmland. Units with a very low ESV had the same distribution as construction land.

The estimated ecosystem service functions and their changes are shown in Table 8. During the last 10 years, the biological control, nutrient cycling, soil formation, water supply, and waste treatment ecosystem service functions have reduced and come to comprise −8.51% of the total ESV. Nutrient cycling recorded the greatest reduction, followed by soil formation and biological control, and waste treatment recorded the lowest. Provisioning services were the greatest contributor of ESV (51% in 2010; 41% in 2020), followed by supporting services (37% in 2010; 35% in 2020) and regulating services (25% in 2010; 30% in 2020).

### 3.4. Analysis of Coefficient of Sensitivty

The coefficient of sensitivity (CS) for all LULC types shown in Table 9 is less than one (1). That means that the assessed total ESV was quite stable and had low sensitivity in response to the value coefficient. The lowest (0.001) and highest (0.686) values of CS were recorded for grazing land and forests, respectively (Table 9). Farmland and shrubland have a relatively low CS. The coefficient of elasticity (CS) of wetland is also important (0.101). Forest land recorded the highest coefficient of CS because it has the largest coverage and is the greatest contributor of ESV in the study area. The overall results show that the coefficient of elasticity (CS) estimated in this study is stable despite the uncertainties that exist in the value coefficient.

## 4. Discussion

### 4.1. Land-Use and Land Cover Changes Impacts on Ecosystem Services Value

**Figure 7:**
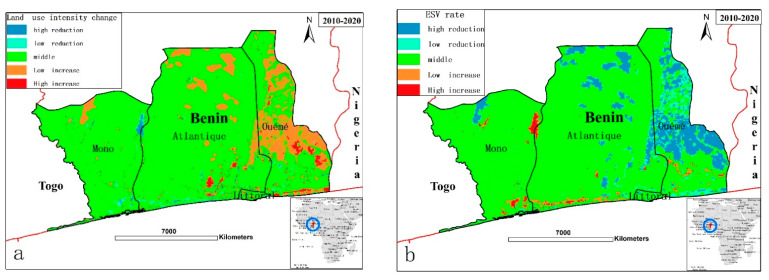
Consistency of land-use intensity changes with the change rate of ESV. (**a**) Map of land-use intensity 2010–2020; (**b**) Map of rate of ESV from 2010–2020.

The spatial distribution of land-use intensity from 2010–2020 was consistent with the spatial distribution of the ESV change rate from 2010–2020 (Figure 7). The ESV of regions with a high land-use intensity index significantly reduced during the 10 years, indicating that the land-use changes influenced ESV significantly under the current urbanizing coastal zone of Benin. Over the last 10 years in our study area, significant changes in LULC patterns have influenced the quality and quantity of ecosystem services, as well as the provision of functions. LULC changes caused changes in the structures and functions of the ecosystem services through the interaction of multiple aspects [40,41].

Between 2010 and 2020, a clear increase in artificial surfaces and farmland and reductions in forest land and waterbodies were recorded. The reductions in forest land and waterbodies are reflected in the fluctuations in total ESV. Traditionally, deforestation along the coastal zone involves clearing land for farming activities, as well as developing settlements and infrastructure. Consequently, the transition of land use is observed between forest and artificial surfaces. The decline in forest area is an important contributor to the total ESV and may have led to the reduction of the provision of ESV.

The reductions in forest land and waterbodies are reflected in the fluctuations in total ESV. Our results cohere with those of previous efforts to quantify the ESV along the coastal zones of Tanzania by Ligatea et al. [38] and Abu Yousuf et al. [42] in Bangladesh’s coastal areas, which revealed that vegetation cover also underwent a net decrease (8.26%). They also showed a reduction of 15.23% in the ESV of the Zhejiang coastal area, due to a 25.13% reduction in forest land [24], from USD 1.33 million to USD 1.07 million due to the loss of vegetation (M. Das et al.) [43]. During our study period, the increased trend of artificial surfaces has contributed to the decline of forest area and waterbodies shrinking. Therefore, our findings are within the existing documentation that many tropical ecosystems suffer from urbanization. These findings imply that locally and globally unplanned urbanization in coastal areas represents a threat to coastal ecosystems.

### 4.2. Sustainable Coastal Zones and Management and Policy Implications

West Africa’s coastal areas, including the Beninese coastal region, face several challenges which put pressure on natural coastal resources and cause the degradation of ecosystem services. Increased human activity has resulted in LULC changes [24,44]. The recent dynamic of coastal development has tended to give rise to extreme LULC changes and the degradation of the ecological environment. A large conversion of forest land and waterbodies into construction land has also taken place.

The valuation of ESV makes key scientific information available for use in decision-making related to coastal zones’ management and the rational use of ecosystem services. Similar to the rest of West Africa’s coastal areas, the coastal zone of Benin sustains various coastal ecosystems that are crucial for local populations’ livelihoods and health, and which require proper management. Sustainable coastal zones and the rational use of coastal resources remain a priority of the local government. However, the total ESV during our study declined from USD 7.1557 million to USD 6.5941 million.

Current strategies face the challenge of the increasing degradation of the coastal ecosystem and the services it provides for local populations, livelihoods, and health. One of the main causes of the degradation of the coastal environment is the growth of the coastal population, which puts pressure on ecosystems and coastal environments. Additionally, the current policies and strategies lack the cooperation and enforcement needed to ensure the sustainable management of the coastal zones and the rational use of the ecosystems. In this context, we should construct effective policies to reduce the loss of ESV and sustain coastal zone management.

The first step is to promote local governance through collaboration between local authorities, institutions, and the population, and thus ensure appropriate decision-making related to coastal ecological environment protection and to provide additional socio-economic benefits to the value of these zones. We must also develop the design of spatial coastal zone management (ISCZM) to integrate LULC changes; coastal development and decision-making should consider ecosystem service losses to ensure that ecosystems are preserved. Further, we should promote the valuation of coastal ecosystem services by stakeholders in order to improve planning decisions and thus ensure successful policy implementation in environmental management and decision-making.

It is important to integrate socio-economic development and biophysical constraints into harmonized ICZM to ensure rational coastal land-use based on the indicators of the sustainable development goals (SDGs).

### 4.3. Limitations of the Study

Due to the limitations of the assessment methods and data availability, the results obtained in this study might include mistakes. One of the major limitations concerns constructing the model empirically with estimates based on the data collected from relevant studies, as this approach assumes the homogeneity of ecosystem service value across all LULC types [17,45,46,47]. The BTA will be accurate if the ESV of an LULC is estimated based on the most similar ecosystems [22,48,49,50]. However, in this case, not all LULC types have corresponding ecosystems. Additionally, the study area, the coastal zone of Benin, was constructed on the local scale, and the areas of natural LULC types, such as forest land and waterbodies, are relatively small, resulting in a lower total ESV.

Despite these limits, the BTA plays a crucial role in ESV assessment in low-income coastal areas such as Benin, where financial constraints remain a challenge to the collection of primary data.

## 5. Conclusions

The present study has analyzed the role of LULC changes in ESV fluctuations within the urbanized tropical coastal zones of Benin in West Africa. Land-use types changed significantly from 2010 to 2020 due to intensification of human activities, proved by the dramatic farmland expansion and increased artificial surfaces which have led to the large shrinkage of grazing land, shrubland and forests. From the above results, the following conclusions have been drawn:

(1) The total ESV during the study period was reduced from USD 7.1557 million in 2010 to USD 6.5941 million in 2020, a reduction of −8.51%. Under the current process of urbanization, the intensity of LULC changes has increased, especially in the eastern central coastal region of Cotonou metropolis and the industrial coastal region of Seme;
(2) Significant changes in specific ecosystem function have been recorded, such as erosion control, climate regulation, biological control, nutrient cycling, soil formation, water supply, and waste treatment. However, provisioning services were the highest contributor to ESVs (51% in 2010; 41% in 2020), followed by supporting services (37% in 2010; 35% in 2020), and regulating services (25% in 2010; 30% in 2020);
(3) Over the past 10 years, the regions with typically high ESVs have shown reduced ESVs, particularly in the coastal industrial zones of Seme. This recent dynamic of lost ESV in the coastal zones of Benin needs to be considered in order to improve the current strategies for the sustainable management of coastal zones, and to preserve the balance between development initiatives and ecosystem health. Given the ongoing coastal population pressure and the intensification of socioeconomic activities in the coastal zone, it is prospective that an increasing demand for land use will place heavy pressure on these ecosystems. Therefore, the capacity of coastal ecosystems to offer ecological functions and services to sustain the life of human beings will be further weakened. Therefore, it is important to regulate and balance population and socioeconomic activities in order to achieve efficient sustainable coastal management; not only must strict provision be made but also training for coastal land use under better monitoring and environmental precautions must be given priority as well as the most efficient technology in the conciliation of environment and economy;
(4) The methodology of this study is economically feasible and has the potential to contribute to policy formulation for data poor areas. Further research in determining the value of the various coastal ecosystems such as coastal wetland could help remote sensing-based assessment of ecosystem value, and is expected to result in sustainable coastal resources management.

## Figures and Tables

**Table 1:** Description of land-use and land cover types.

LULC Types	Description
Forest land	Over 30% of land covered with trees and vegetation
Grazing land	Over 10% of land covered with natural grass
Shrubland	Land covered with woody perennial shrubs.
Farmland	Cropland is used for agriculture, horticulture, and gardens, including paddy fields and irrigated and dry farmland.
Wetland	Plants and waterbodies, including inland marshes and lakes.
Waterbody	Waterbodies in the area, including rivers, lakes, reservoirs, and fishponds
Artificial surfaces	Land modified by human activity for settlements, as well as industrial and mining activity.

**Table 2:** Stages of measurement of land-use intensity.

Intensity Level	LULC Types Classification	Value
Light land-use increase	Wetland	1
Low land-use increase	Waterbody	2
Middle land-use increase	Forest land, grazing land, shrubland	3
Strong land-use increase	Farmland	4
High land-use increase	Artificial surfaces	5

**Table 3:** Land cover and land-use types with their ecosystem service value coefficient equivalents (USD/ha/).

LCLU Type Composition Equivalent Biome				Coefficient Value (USD/Ha/Year)	
				Global Value	Local Value
Forest	Forest land, open forest land		Forest	96,900	1093.200
Grazing land	Moderate coverage grassland	Grassland	Grasslands	23,200	355.500
Shrubland	Grass/grasslands		Woody perennial plants, >0.5 m and <5 m	23,200	89700
Farmland	Paddy fields, maize, and sesame fields		Cropland	9200	169.200
Wetland	Wetland plants and water bodies		Wetland	1,478,500	2856.100
Waterbody	Rivers, land reservoirs, fisheries, and lakes		Lakes/rivers	849,800	3226.800
Artificial surfaces	Residential, commercial,	Settlements and roads	Urban	000	000

**Table 4:** Ecosystem service functions and their modified local value coefficients (USD/ha/year).

Ecosystem Services	Subtypes	Farmland	Forest	Shrubland	Grazing Land	Waterbody	Wetland
Provisioning services	Water supply		8	8		2117	130.19
	Food production	187.57	32	32	117.45		185.68
	Raw material		51.2	51.2			151
	Genetic resources		41	41			49.42
	Medical services						71.17
Regulating services							
	Waste treatment		136	136	87	431.5	23.84
	Erosion control		245	245	29		58.74
	Climate regulation		223	223			143.99
	Biological control	24				23	
	Gas regulation		13.68	13.68		7	48.7
	Disturbance regulation		5	5			
Supporting services							
	Nutrient cycling		184.4	184.4	25		74.06
	Pollination	14	7.27	7.27			
	Soil formation		10	10	1		31.43
	Habitat/refugia		17.3	17.3			496.64
Cultural services	Recreation		4.8	4.8	0.8	69	14.96
	Cultural		2	2			47.68
Total		986.69	293.25	2063.53	293.25	8103.5	2063.53

**Table 5:** Changes in land-use and land cover in Benin tropical coastal area from 2010 to 2020.

Years	2010	2020	2010–2020	
LULC Types	Area (10^6^ ha)		Variation	
			Area (10^6^ ha)	Rate/%
Farmland	74.76	705	630.29	63%
Forest	4795.9	4136	−659.9	−65.99
Grazing land	31.23	21.11	−10.11	−1.01%
Shrubland	335.96	209.2	−126.8	−3.7%
Wetland	164.57	234.8	70.22	4.20%
Waterbody	345.51	336.4	−9.15	−0.92%
Artificial	302.27	407.8	105.53	10.55%

**Table 6:** Conversion matrix of land-use types in Benin tropical coastal zones from 2010 to 2020.

Area/km^2^		Year 2020									
		Farmland	Forest	Grazing Land	Shrubland	Wetland	Waterbody	Artificial Surfaces	Total Area 2020	Loss	Change Rate/%
**Year 2010**	Farmland	**61.27**	0.50	0.02	0.15	0.46	0.12	12.23	74.75	13.47	843.06%
	Forest	577.54	**4097.89**	0.55	22.32	36.66	9.51	51.24	4795.72	4218.17	−1.73%
	Grazing land	0.89	0.54	**16.55**	1.24	0.79	0.07	10.92	31.20	30.11	−29.05%
	Shrubland	63.63	23.31	1.19	**182.81**	25.51	1.63	37.82	335.93	272.28	−18.85%
	Wetland	0.26	1.52	2.23	0.67	**151.59**	4.98	3.29	164.54	164.28	27.16%
	Waterbody	0.39	5.53	0.06	0.77	17.66	**319.84**	1.23	345.47	345.09	−2.57%
	Artificial Surfaces	0.95	6.55	0.47	1.15	1.98	0.18	**290.88**	302.25	301.22	31.16%
	Gain	643.66	4135.35	21.05	208.96	234.19	336.21	395.38	/	/	/
	Total Area 2010 Change direction	704.96 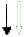	4135.85 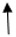	21.09 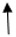	209.13 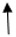	234.76 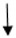	336.33 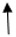	407.77 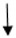	6051.34	/	/

**Table 7:** Fluctuations in ecosystem service value between 2010 and 2020.

LULC	2010	2020	ESV Change/10^6^ USD	
Types			2010–2020	Change Rate%
Farmland	0.0127	0.1194	0.1067	843.17%
Forest	5.2458	4.5239	−0.7219	−13.76%
Grazing land	0.011	0.0074	−0.0036	−32.56%
Shrubland	0.3014	0.1876	−0.1138	−37.76%
Wetland	0.4698	0.6704	0.2006	42.70%
Waterbody	1.1149	1.0854	−0.0295	−2.65%
Artificial surfaces	0000	0000	0000	
Total	7.1557	6.5941	−0.5615	−7.85%

**Table 8:** Changes in ecosystem service function (USD million per year).

		ESV_f_ (Million USD)		Value	Change Rate %
Ecosystem Services		2010	2020	2010–2020	
Provision	Water supply	0.79	0.77	−0.02	−25
	Food production	0.25	0.33	0.08	32
	Raw material	0.28	0.25	−0.03	−1.071
	Genetic resources	0.21	0.18	−0.03	−14.28
	Medical services	0.11	0.16	0.05	45.45
Regulating	Water regulation	0.2	0.19	−0.01	−5
	Waste treatment	0.87	0.74	−0.13	−14.94
	Erosion control	0.12	0.1	−0.02	−16.66
	Climate regulation	0.11	0.1	−0.01	−9
	Biological control	0.97	0.24	−0.73	−75
	Gas regulation	0.8	0.73	−0.07	−8.75
	Disturbance regulation	0.25	0.21	−0.04	−16
Supporting	Nutrient cycling	0.95	0.81	−0.95	−100
	Pollination	0.54	0.5	−0.04	−7
	Soil formation	0.56	0.5	−0.54	−96.24
	Habitat/refugia	0.17	0.19	0.02	11
Cultural	Recreation	0.51	0.47	−0.04	−7.84
	Cultural	0.19	0.2	0.01	5.26
	Total	0.83	0.7	−0.13	−15

**Table 9:** Analysis of coefficient of elasticity.

LCLU Types	Year 2010		Year 2020	
	ESV/10^6^ USD	CE	ESV/10^6^ USD	CE
Farmland	0.012	0.001	0.119	0.018
Forest	5.245	0.733	4.523	0.686
Grazing land	0.011	0.001	0.007	0.001
Shrubland	0.301	0.042	0.187	0.028
Wetland	0.469	0.065	0.670	0.101
Waterbody	1.114	0.155	1.085	0.164
Artificial surface	0000	0000	0000	0000

## Data Availability

Not applicable.
